# Salivary Calculus

**Published:** 1907-06-08

**Authors:** 


### Points in Surgery.

### SALIVARY CALCULUS.

Salivary calculus occurs most commonly in the submaxillary gland. The numerous simple glands which are distributed throughout the buccal mucosa are also apt to become plugged by little white particles which may be regarded as minute calculi. The sublingual is not often affected; the parotid practically immune. This immunity is to be attributed to the nature of the parotid secretion, and perhaps also to the position of the orifice of its duct, which is placed unfavourably for the entrance of foreign substances.

### Causes of Salivary Calculus.

Often a foreign body, for example the bristle of a tooth-brush, forms the nucleus of a concretion, and may be regarded as the source of the trouble. In other cases no such nucleus is discovered; to explain these, acidity of the saliva has been suggested, and cases have been recorded where salivary calculus has been said to be associated with acidity of the saliva. This has not been the writer's experience. But there is a simple fallacy in testing the reaction of the saliva; for it will turn blue litmus red and red litmus blue. If only blue litmus paper be used, the result may lead the observer to the erroneous belief that the saliva is acid. However, it seems reasonable to suppose that the local production of acidity in the submaxillary duct, such as might occur through the action of micro-organisms, would lead to the deposition of a concretion from the mucous-laden secretion of the gland.

Turning to the practical side of the matter, the simple buccal glands may be dismissed in a few words. They may be compared with the sebaceous glands of the skin. Just as the duct of a sebaceous gland may become blocked and a sebaceous cyst be formed, so may the duct of a simple salivary gland become blocked and a salivary cyst be produced. On the lips and cheek such cysts do not attain large size, probably because they become ruptured by the teeth; but in the floor of the mouth they may attain a diameter of one inch. The treatment of these simple salivary cysts is to apply a local anæsthetic to the overlying mucous membrane, and snip away a portion of this with scissors.

### Submaxillary Calculus.

The symptoms of submaxillary calculus are due to blocking of Wharton's duct. Usually the patient will give a history of a submaxillary swelling, which gets larger and more tender during and after meals, and diminishes in size or perhaps disappears entirely before the next meal. Sometimes he will complain that he can feel something wrong in the floor of the mouth, underneath the tongue. Inspection of the floor of the mouth may reveal some abnormality of the papilla and orifice of the duct, which are situated near the frænum of the tongue. Often the mouth of the duct is occupied by a small plug of putty-like material; and the papilla in which the duct terminates may be swollen, so that instead of being flaccid and indistinct like its fellow on the healthy side, it stands up rigid and prominent. Sometimes there is petechial hæmorrhage in the papilla. Palpation, by means of one finger in the mouth and a finger of the other hand beneath the jaw, may reveal a calculus lying in the duct, and close beneath the mucous membrane; and occasionally, but not often, pressure in this region will cause pus to exude from the duct. On the other hand, the calculus may be situated deep in the gland, so that it cannot be felt by palpation. In such a case a probe may be passed along the duct in the following manner, and the calculus felt. The mucous membrane over and around the papilla is swabbed with a 5 per cent. solution of cocaine. When this has taken effect, the papilla is steadied with a pair of conjunctival fixation forceps, and a fine sterilised lachrymal probe is inserted and gently passed backwards along the duct. But the patient's history, together with inspection of the papilla and palpation of the floor of the mouth, as a rule will yield all the information that is required, and, apart from the discomfort caused to the patient, the passage of a probe may fail to detect a calculus, even when one is present, and it is further open to the objection that by its introduction sepsis may be started. This is an important point, for a gland whose duct is blocked by a calculus is prone to suppurate. In untreated cases, one of two events may come about. Either the gland will in time cease to secrete and become converted into an indurated mass, a spontaneous cure being the result, or suppuration may ensue. And suppuration in the submaxillary region, especially if its cause be unrecognised and the calculus be allowed to remain, is apt to have unhappy results, which it will not be necessary to consider in detail.

### Treatment.

If the calculus be in the duct and close under the mucous membrane, it may be removed under cocaine anæsthesia, through an incision of the overlying buccal mucosa—an operation which is not always so painless as it sounds. But if suppuration has occurred in the gland, or if the calculus is too deeply situated for removal through the mouth, an external wound is necessary. In the presence of an abscess it will be necessary merely to open the abscess, insert a finger in order to find and remove the calculus, and then to effect drainage. In cases which are not septic the indurated submaxillary gland had better be dissected out entire with its contained calculus. That is effected most easily by a curved incision below the jaw, something similar to the text-book incision for ligature of the lingual artery. A large vein—the temporo-facial—will be divided; otherwise there are no especial difficulties in the operation.

